# Liver Transplantation in the Treatment of Bleeding Oesophageal Varices

**DOI:** 10.1155/1989/57895

**Published:** 1989

**Authors:** Layton F. Rikkers

**Affiliations:** Department of Surgery University of Nebraska Medical Center 42nd and Dewey Avenue Omaha Nebraska 68105 USA


### REFERENCES

1. Ranson, J.H.C. (1979) The timing of biliary tract surgery in acute pancreatitis. *Ann. Surg*; **189**: 654–663.
2. Semel, L., Schrieber, D., Fromm, D. (1983) Gallstone pancreatitis. *Archiv. Surg*; **118**: 901–904.
3. Tondelli, P., Stutz, K., Harder, F., Schuppisser, J-P., Allgower, M. (1982) Acute gallstone pancreatitis: best timing for surgery. *Br. J. Surg;* **69**, 709–710.
4. Kim, U., Shen, H-Y., Bodner, B. (1988) Timing of surgery for actue gallstone pancreatitis. *Am. J. Surg;* **156**: 393–396.
5. Stone, H.H., Fabian, T.C., Dunlop, W.E. (1981) Gallstone pancreatitis. *Ann. Surg;* **195**: 305–312.
6. Safrany, L., Cotton, P.B. (1981) A preliminary report: urgent duodenoscopic sphincterotomy for acute gallstone pancreatitis. *Surgery:* **89**: 424–428.
7. Seigal, J.H., Tone, P., Menikeim, D. (1986) Gallstone pancreatitis: pathogenesis and clinical forms — the emerging role of endoscopic management. *Am. J. Gastroenterol*; **81**: 774–778.
8. Belghiti, J., Kleinman, P., Cherqui, D., Pernceni, T., Bernades, P., Fekete, F. (1987) Traitement precoce de la lithiase biliaire au cours des pancreatites biliaires. *Gastroenterol. clin Biol*; **11**: 786–789.
9. Rosseland, A.R., Solhaug, J.H. (1984) Early or delayed endoscopic papillotomy in gallstone pancreatitis. *Ann. Surg*; **199**: 165–167.
10. Neoptolemos, J.P., London, N. Slater, N.D., Carr-Locke, D.C., Fossard, D.P., Moosa, A.R. (1986) A prospective study of ERCP and endoscopic sphincterotomy in the diagnosis and treatment of gallstone acute pancreatitis. *Archiv. Surg*; **121**: 697–702.
11. Neoptolemos, J.P., Carr-Locke, D.L., London, N., Bailey, I. Fossard, D.P. (1988) ERCP findings and the role of endoscopic sphincterotomy in acute gallstone pancreatitis. *Br. J. Surg*; **75**: 954–960.
12. Sawfey, H., Bulkley, G.B., Cameron, J.L. (1984) The role of oxygen-derived free radicals in the pathogenesis of acute pancreatitis. *Ann. Surg;* **200**: 405–413.

### LIVER TRANSPLANTATION IN THE TREATMENT OF BLEEDING OESOPHAGEAL VARICES

### ABSTRACT

*S. Iwatsuki, T.E. Starzl, S. Todo, R.D. Gordon, A.G. Tzakis, J.W. Marsh, L. Makowka, B. Koneru, A. Stieber, G. Klintmalm, B. Husberg, D. van Thiel. (1988) Liver transplantation in the treatment of bleeding esophageal varices. Surgery, 104; 697–705.*

**From March 1980 to July 1987, 1000 patients with various end-stage liver diseases received orthotopic liver transplants. Of the 1000 patients, three hundred two had definite histories of bleeding from esophageal varices before transplantation. There were 287 patients with nonalcoholic liver diseases and 15 patients with alcoholic cirrhosis. All patients had very poor liver function, which was the main indication for liver transplantation. One- through 5-year actuarial survival rates of the 302 patients were 79%, 74%, 71%, 71%, and 71%, respectively. These survival rates are far better than those obtained with other available modes of treatment for bleeding varices when liver disease is advanced. Long-term sclerotherapy is the treatment of primary choice for bleeding varices. Patients in whom sclerotherapy fails should be considered for liver transplantation unless clear contraindications exist.**

KEYWORDS: Liver transplantation; sclerotherapy; portal hypertension; portosystemic shunts

**PAPER DISCUSSION**

In the manuscript under review, Iwatsuki and associates[1] have retrospectively evaluated 1,000 patients undergoing orthotopic liver transplantation at the University of Colorado, University of Pittsburgh, and the Pittsburgh affiliated Baylor University Medical Center in Dallas between March 1980 and July 1987. All of these patients were transplanted in the cyclosporine-era, during which results have been considerably better than those achieved prior to 1980. Although all transplants were done for end-stage liver disease, a review of the pre-transplant courses revealed that 302 patients had bled from varices. For reasons that are not clear, the group of patients who had experienced variceal hemorrhage had significantly better survival after transplantation than had those patients without a history of variceal bleeding.

Variceal hemorrhage had been treated by endoscopic sclerotherapy in 219 patients, by portal-systemic shunt in 37 patients (nonselective = 22, selective = 15), and by a devascularization, nonshunt procedure in five patients. It is not specified in the manuscript whether any of the previously operated patients had also undergone sclerotherapy. Only ten individuals (3%) were bleeding from varices in the immediate pre-transplant interval. Kaplan–Meier analysis showed no significant difference in survival between the 42 patients who had undergone prior surgery for variceal hemorrhage and the 260 patients who had received only sclerotherapy or no treatment for variceal bleeding, although the former group had a slightly higher one-month mortality (17% versus 8%).

The authors show that their survival results of transplant patients with prior variceal bleeding compare favorably to several other published series of shunt surgery, nonshunt operations, and sclerotherapy; and that survival after transplantation is clearly superior to survival data from other series of variceal bleeders when only Child's C patients are included in the analysis. Iwatsuki and co-workers conclude that long-term sclerotherapy is the preferred treatment for variceal hemorrhage and that, unless clear contraindications exist, hepatic transplantation should be done if and when sclerotherapy fails or end-stage liver disease develops. In other words, they state that non-transplant surgery for bleeding varices is rarely, if ever, indicated. Are these conclusions justified from the data presented?

I believe that the authors' conclusions are appropriate for the population of patients included in their investigation. All of these patients apparently had end-stage liver disease and were reasonable candidates for liver transplantation. However, these are highly selected, referred patients with almost exclusively nonalcoholic liver diseases. In addition, a significant percentage of the patients were in the pediatric age range. Therefore, it is inappropriate to compare the survival of these patients after transplantation with the predominantly adult, alcoholic cirrhotic patients included in the several reports referred to in the discussion section of their manuscript. Additionally, the patients who comprised these various series presented with the problem of variceal hemorrhage and were specifically treated either emergently or electively for that complication of portal hypertension. In contrast, only 3% of the variceal bleeders in Iwatsuki's report were actively bleeding at the time of transplantation.

In addition to hepatic transplantation, there are available to the physician or surgeon pharmacotherapy, sclerotherapy, and a large variety of shunt and nonshunt operations when treating patients with variceal hemorrhage in 1989. Transplantation is the only one of these therapies that addresses the underlying liver disease in addition to preventing recurrent bleeding. Considering the excellent post-transplantation longterm survival rates, which have been demonstrated in several centers throughout the world, few would argue with the concept that transplantation is the preferred treatment for many patients with marginal hepatic functional reserve or a poor quality of life secondary to their liver disease, whether or not they have bled from varices. Unfortunately, many variceal bleeders are not appropriate transplant candidates and, even if they were, the supply of donor organs would never meet the demand. The most common causes of variceal hemorrhage world-wide are schistosomiasis, a disease in which hepatic functional reserve is often indefinitely maintained, and alcoholic cirrhosis, an affliction often accompanied by dysfunction of other major organ systems and/or incurable alcoholism. Although carefully selected patients with alcoholic cirrhosis have been successfully transplanted[2], it is unlikely that this treatment will ever be applicable to the majority of patients with this disorder. Likewise, several patients with nonalcoholic liver disease are not transplant candidates because of age, coexisting diseases, or financial resources. Therefore, other means of preventing recurrent hemorrhage in patients with portal hypertension need to be considered.

Recent controlled trials[3–5] of sclerotherapy versus the distal splenorenal shunt suggest that endoscopic variceal sclerosis is a reasonable initial therapy for most cirrhotics with variceal hemorrhage. Exceptions include noncompliant patients, those living at great distances from tertiary medical care, and individuals who bleed from gastric varices or portal hypertensive gastropathy[4]. Unless these latter patients present with marginal hepatic functional reserve and are otherwise reasonable transplant candidates, a portal-systemic shunt or nonshunt operation needs to be done to prevent exsanguination. In addition, approximately one-third of patients managed with long-term variceal sclerosis will eventually fail this mode of therapy and require another treatment to prevent recurrent hemorrhage[3,4]. One trial[3] has shown that most sclerotherapy failures can be salvaged by shunt surgery while a second study[4] including more noncompliant patients living in geographically remote areas, showed a lower salvage rate for sclerotherapy failures.

Although Iwatsuki implies in the discussion of his results that patients transplanted without prior surgery for portal hypertension survived longer than those who had undergone portal systemic shunts prior to transplantation, his own analysis of these two groups showed no significant differences in survival. The survival of previously shunted patients might have been even better if subsequent transplantation had been considered at the time of the initial operation. Although the specific types of shunts are not listed, presumably many of the nonselective shunts were of the portacaval variety, which make subsequent hepatic transplantation a much more challenging technical feat. Others[6] have suggested that, when portal-systemic shunt surgery is necessary in patients who may be future candidates for transplantation, the porta hepatis should be avoided and either a distal splenorenal shunt or an interposition mesocaval shunt constructed. I prefer selective variceal decompression for most patients because hepatic portal perfusion is maintained in a significant percentage and analysis of all of the controlled data available suggests that postoperative encephalopathy is less frequent following selective shunts than after nonselective shunting procedures[7].

In conclusion, hepatic transplantation is appropriate therapy for some variceal bleeders either as initial treatment or after they have failed sclerotherapy. However, patients who fail sclerotherapy and are either not candidates for transplantation or who have slowly progressive hepatocellular disease with good functional reserve should undergo selective or nonselective portal decompression or a nonshunting operation depending on each individual's hemodynamic status and clinical circumstances[8]. The only meaningful way to compare these various treatment schemes in a clinical investigation would be to include only potential transplant candidates at the outset. Ideally, such a study would be randomized with one arm including patients who either underwent initial hepatic transplantation or transplantation after sclerotherapy failure and the second arm consisting of individuals who would receive a shunt operation after sclerotherapy if their hepatic functional reserve was well maintained. This group would then undergo transplantation as a back-up to shunt surgery when end-stage liver disease was apparent. Because many variceal bleeders are not candidates for hepatic transplantation, it would be difficult to accrue a sufficient number of patients to such an investigation to provide meaningful results.

Layton F. Rikkers, M.D.
Professor and Chairman, Department of Surgery
University of Nebraska Medical Center
42nd and Dewey Avenue
Omaha, Nebraska 68105

### REFERENCES

1. Iwatsuki, S., Starzl, T.E., Todo, S., Gordon, R.D., Tzakis, A.G., Marsh, J.W., Makowka, L., Koneru, B., Stieber, A., Klintmalm, G., Husberg, B., van Thiel, D. (1988) Liver transplantation in the treatment of bleeding esophageal varices. *Surgery*; **104**: 697–705.
2. Starzl, T.E., van Thiel, D., Tzakis, A.G., Iwatsuki, S., Todo, S., Marsh, J.W., Koneru, B., Staschak, S., Stieber, A., Gordon, R.D. (1988) Orthotopic liver transplantation for alcoholic cirrhosis. *JAMA*; **260**: 2542–2544.
3. Warren, W.D., Henderson, J.M., Millikan, W.J., Galambos, J.T., Brooks, W.S., Riepe, S.P., Salam, A.A., Kutner, M.H. (1986) Distal splenorenal shunt versus endoscopic slcerotherapy for long-term management of variceal bleeding. *Ann. Surg*; **203**: 454–462.
4. Rikkers, L.F., Burnett, D.A., Volentine, G.D., Buchi, K.N., Cormier, R.A. (1987) Shunt surgery versus endoscopic sclerotherapy for long-term treatment of variceal bleeding. *Ann. Surg*; **206**: 261–271.
5. Teres, J., Bordas, J.M., Bravo, D., Visa, J., Grande, L., Garcia-Valdecasas, J.C., Pera, C., Rodes, J. (1987) Sclerotherapy versus distal splenorenal shunt in the elective treatment of variceal hemorrhage: a randomized controlled trial. *Hepatology*; **7**: 430–436.
6. Brems, J.J., Hiatt, J.R., Klein, A.S., Millis, J.M., Colonna, J.O., Quinones-Baldrich, W.J., Ramming, K.P., Busuttil, R.W. (1989) Effect of a prior portasystemic shunt on subsequent liver transplantation. *Ann. Surg*; **209**: 51–56.
7. Rikkers, L.F. (1988) Is the distal splenorenal shunt better? *Hepatology*; **8**: 1705–1707.
8. Rikkers, L.F., Soper, N.J., Cormier, R.A. (1984) Selective operative approach fr variceal hemorrhage. Am. J. Surg; **147**: 89–96.

### SURGERY OR ENDOPROSTHESIS FOR MALIGNANT OBSTRUCTIVE JAUNDICE

### ABSTRACT

*Shepherd HA, Royle G, Ross APR, Diba A, Arthur M and Colin-Jones D (1988) Endoscopic biliary endoprosthesis in the palliation of malignant obstruction of the distal common bile duct: a randomized trial. British Journal of Surgery; 75:1166–1168*

**A total of 52 jaundiced elderly patients who had malignant obstruction of the distal common bile duct and who required palliative biliary decompression were randomized to**

